# Prevalence and Factors Associated with Tinnitus: A Community-Based Study of Japanese Elders

**DOI:** 10.2188/jea.JE20090121

**Published:** 2010-07-05

**Authors:** Takehiro Michikawa, Yuji Nishiwaki, Yuriko Kikuchi, Hideyuki Saito, Kunio Mizutari, Michiko Okamoto, Toru Takebayashi

**Affiliations:** 1 Department of Preventive Medicine and Public Health, School of Medicine, Keio University, Tokyo, Japan; 2 Department of Otorhinolaryngology, Head and Neck Surgery, School of Medicine, Keio University, Tokyo, Japan; 3 Haruna and Kurabuchi Health Center, Takasaki City Hall, Takasaki, Japan

**Keywords:** tinnitus, prevalence, risk factors, aged

## Abstract

**Background:**

The prevalence of tinnitus is reported to be high in older Western populations, and several risk factors have been suggested. However, community-based evidence on prevalence is limited and, to our knowledge, there is no such information from older non-Western populations. The purpose of this study was to determine the prevalence and factors associated with tinnitus in community-dwelling Japanese elders.

**Methods:**

In this community-based cross-sectional study, 1320 residents of Kurabuchi Town aged 65 years or older (584 men and 736 women; participation proportion = 98.7%) were interviewed at home in 2006, and information on tinnitus and factors associated with tinnitus was collected. We estimated the prevalence of tinnitus by age group and sex and used a logistic regression model to investigate associated factors.

**Results:**

The prevalence of tinnitus was 18.6% (men: 18.0%; women: 19.0%); there were no statistically significant differences by age group or sex. Hearing difficulty, depressive mood, prescribed medication, past/current history of coronary heart disease, and knee joint pain requiring medical consultation were associated with tinnitus.

**Conclusions:**

These findings suggest that tinnitus is common in Japanese aged over 65 years. Because the factors associated with tinnitus in this cross-sectional study are potentially modifiable, they should be thoroughly investigated in a longitudinal study.

## INTRODUCTION

Tinnitus is the perception of sound in the absence of an apparent acoustic stimulus; its pathological mechanisms and clinical features are still not fully understood.^1^^–^^3^ The prevalence of tinnitus is reported to be relatively high in older populations, but results vary widely among studies (from 7.6% to 30.3% in Western populations aged 50 years or older)^1^^,^^4^^,^^5^ due to differences in the populations studied and methodologies used. Because most community-dwelling individuals with ear problems do not seek help,^6^ community-based studies with high response proportions are necessary to determine the true prevalence of tinnitus. Unfortunately, there have been few such studies.^4^^,^^5^ In addition, there is a lack of evidence regarding age trends in tinnitus prevalence in older populations,^1^^,^^2^ and information on sex differences in prevalence remains inconclusive.^4^^–^^10^

Studies have shown that tinnitus can reduce quality of life and lead to negative well-being in older populations.^11^^,^^12^ There is no consensus regarding optimal treatment,^3^ so prevention is important. The reported risk factors include hearing impairment, noise exposure, head and neck injuries, medication, ear diseases, cardiovascular diseases, lifestyle factors, and mental status^1^^,^^2^; however, most studies have been clinic/hospital-based or have investigated younger populations.^13^ Data from community-based samples are very limited,^4^^,^^13^ and, to our knowledge, there are none at all from older non-Western populations.

The aims of this cross-sectional study were to determine the prevalence and factors associated with tinnitus in community-dwelling Japanese elders. Great effort was made to obtain data that were highly representative of our target population.

## METHODS

### Study population

This community-based cross-sectional study was carried out in Kurabuchi Town, a rural part of Takasaki City, Gunma Prefecture, which is located about 100 km north of Tokyo. It is part of the Kurabuchi Study,^14^^–^^16^ an ongoing longitudinal study of sensory impairments and orthopedic symptoms. Using resident registration records, the local municipal government made a list of all residents of Kurabuchi who were aged 65 years or older. Based on this list, the data for this study were collected in 2006 during home-visit health interviews, during which a structured questionnaire was used by trained public health nurses and local welfare commissioners to collect subjective health information. A total of 1337 eligible residents—excluding those institutionalized or hospitalized—were identified, and 1320 residents (584 men and 736 women; 98.7% of those eligible) answered 2 questions on tinnitus.

This study was approved by the Ethics Committee of the School of Medicine, Keio University (Tokyo, Japan). The anonymous data were received directly from Takasaki City.

### Assessment of tinnitus

Because there are no standardized questions for eliciting information on tinnitus,^4^^,^^11^ we used modified questions from earlier studies.^4^^,^^5^^,^^17^ The subjects were asked, “In the past year have you experienced any ringing, buzzing, or other sounds (tinnitus) in your ears?” The response options were “Yes” and “No”. Those who responded “Yes” were asked an additional yes/no question: “Have these sounds interfered with your concentration or ability to sleep?”. Those who answered the second question with “Yes” were classified as having severe tinnitus, and those who answered “No” were classified as having mild tinnitus.

### Assessment of associated factors

We collected information on factors associated with tinnitus. Hearing difficulty was evaluated using a question from a large-scale intervention study in the United Kingdom,^18^ which was translated and then back-translated for our study: “Do you have difficulty hearing and understanding what a person says to you in a quiet room if they speak normally to you (even when wearing your hearing aid)?”. The response options were “No” and “Yes”. Other factors assessed were educational level (high school or higher/elementary or junior high), living situation (living alone or with spouse/family/others), smoking status (never/ex/current smoker), alcohol drinking (never/ex/current drinker), and hearing aid usage (no/yes). Depressive mood was evaluated using the question “Do you feel sad, depressed, or miserable?”,^18^ and the possible responses were “no”, “sometimes”, “often”, and “always”. Information on the total number of prescribed medications was also obtained, and then categorized into 2 groups: no or yes (ie, 1 or more prescribed medications). For the abovementioned factors, we inquired as to the participant’s state at the time of interviews. In addition, participants were asked about past/current histories of stroke, coronary heart disease (angina or myocardial infarction), hypertension, diabetes mellitus, and cancer. Studies have shown that data collected through self-reporting of medical conditions are almost as reliable as those obtained from medical records, even in older populations.^19^^,^^20^ Knee joint pain in the last year was evaluated by using 4 categories (no, sometimes, often, and always). If participants answered “sometimes,” “often,” or “always,” they were asked if they had sought medical consultation for their knee joint pain (yes/no). Knee joint pain was then classified into 3 categories: no pain, pain without medical consultation, or pain requiring medical consultation.

### Statistical analysis

The prevalence of tinnitus according to age group (65–69, 70–79, ≥80 years) and sex was calculated; the chi-square test was used to examine sex differences in overall prevalence and differences in prevalence by age group. Age group trends were evaluated with the Cochran–Armitage trend test.

To examine the association between tinnitus and factors associated with tinnitus, subjects were categorized as having mild or severe tinnitus. However, because the number of subjects with severe tinnitus was very small, the groups were instead combined. First, we performed univariate analyses. Then, all of the associated factors identified in the univariate analyses (*P* < 0.2) were included in multivariate analyses using a logistic regression model, and odds ratios (ORs) and 95% confidence intervals (CIs) for tinnitus were calculated. Although age group and sex were not associated with tinnitus, we added them to the multivariate-adjusted model as a priori confounding factors. The fit of the multivariate-adjusted model was assessed by using the Hosmer–Lemeshow goodness-of-fit test (*P* = 0.33).^21^ In addition, we repeated the same analysis after excluding hearing aid users, because hearing aids are sometimes used as a treatment for tinnitus.

The statistical package STATA version 9 (STATA Corporation, College Station, Texas, US) was used to perform all analyses.

## RESULTS

Table 1 summarizes the prevalence of tinnitus by age group and sex. In the total study population, the prevalence was 15.5% (95% CI, 13.6–17.6) for mild tinnitus, 3.0% (2.2–4.1) for severe tinnitus, and 18.6% (16.5–20.8) for any tinnitus (mild + severe). No age group trends or sex differences were observed. Table 2 shows the associations between tinnitus and factors associated with tinnitus. In the univariate analyses, educational level, hearing difficulty, depressive mood, prescribed medication, past/current history of coronary heart disease, and knee joint pain were associated with tinnitus (*P* < 0.2). These 6 factors were included in our multivariate-adjusted model, in addition to age group and sex. As compared with those subjects with no hearing difficulty, the multivariate-adjusted OR for those with hearing difficulty was 1.66 (95% CI, 1.12–2.47). Those with a depressive mood had an increased odds of tinnitus (multivariate-adjusted OR, 1.57; 95% CI, 1.13–2.18). The multivariate-adjusted OR in subjects who were taking prescribed medication was 1.50 (95% CI, 1.02–2.20), as compared with those not taking such medication. A past/current history of coronary heart disease was associated with tinnitus (multivariate-adjusted OR, 1.58; 95% CI, 1.02–2.43), as was knee joint pain requiring medical consultation (1.51; 1.05–2.19). In the subgroup analysis—in which hearing aid users were excluded—the results did not substantially differ.

**Table 1.** Prevalence of tinnitus by age group and sex in Japanese elders

	Age group (years)	No. at risk	Mild tinnitus		Severe tinnitus		Tinnitus (mild + severe)	
		
			*n*	Prevalence (95% CI)	*n*	Prevalence (95% CI)	*n*	Prevalence (95% CI)
Men	65–69	114	16	14.0 (8.2–21.8)	2	1.8 (0.2–6.2)	18	15.8 (9.6–23.8)
	70–79	303	46	15.2 (11.3–19.7)	7	2.3 (0.9–4.7)	53	17.5 (13.4–22.2)
	80–	167	28	16.8 (11.4–23.3)	6	3.6 (1.3–7.7)	34	20.4 (14.5–27.3)
	Total	584	90	15.4 (12.6–18.6)	15	2.6 (1.4–4.2)	105	18.0 (14.9–21.3)

Women	65–69	134	16	11.9 (7.0–18.7)	4	3.0 (0.8–7.5)	20	14.9 (9.4–22.1)
	70–79	331	55	16.6 (12.8–21.1)	12	3.6 (1.9–6.2)	67	20.2 (16.0–25.0)
	80–	271	44	16.2 (12.1–21.2)	9	3.3 (1.5–6.2)	53	19.6 (15.0–24.8)
	Total	736	115	15.6 (13.1–18.5)	25	3.4 (2.2–5.0)	140	19.0 (16.2–22.0)

All	65–69	248	32	12.9 (9.0–17.7)	6	2.4 (0.9–5.2)	38	15.3 (11.1–20.4)
	70–79	634	101	15.9 (13.2–19.0)	19	3.0 (1.8–4.6)	120	18.9 (15.9–22.2)
	80–	438	72	16.4 (13.1–20.2)	15	3.4 (1.9–5.6)	87	19.9 (16.2–23.9)
	Total	1320	205	15.5 (13.6–17.6)	40	3.0 (2.2–4.1)	245	18.6 (16.5–20.8)

Abbreviation: CI, confidence interval.

Note: There were no age group trends on the Cochran–Armitage trend test or sex differences on the chi-square test.

**Table 2.** Factors potentially associated with tinnitus in Japanese elders

	Prevalence of tinnitus (%)^a^	*P*-value^b^	Multi-adjusted OR (95% CI)^c^	*P*-value
Age group				
65–69	38/248 (15.3)		1.00	
70–79	120/634 (18.9)		1.01 (0.66–1.55)	0.973
80–	87/438 (19.9)	0.322	0.91 (0.57–1.45)	0.698
Sex				
Women	140/736 (19.0)		1.00	
Men	105/584 (18.0)	0.629	1.10 (0.81–1.50)	0.523
Education				
High school or higher	43/276 (15.6)		1.00	
Elementary or junior high	198/1020 (19.4)	0.147	1.22 (0.83–1.79)	0.316
Living alone				
Yes	29/139 (20.9)			
No	214/1170 (18.3)	0.461		
Smoking status				
Never smoker	186/1009 (18.4)			
Ex-smoker	25/122 (20.5)			
Current smoker	32/180 (17.8)	0.825		
Alcohol drinking				
Never drinker	158/852 (18.5)			
Ex-drinker	14/54 (25.9)			
Current drinker	69/394 (17.5)	0.329		
Hearing difficulty				
No	187/1105 (16.9)		1.00	
Yes	51/183 (27.9)	<0.001	1.66 (1.12–2.47)	0.011
Depressive mood				
No	161/1002 (16.1)		1.00	
Depressed	82/308 (26.6)	<0.001	1.57 (1.13–2.18)	0.007
Prescribed medication				
No	44/342 (12.9)		1.00	
Yes	198/968 (20.5)	0.002	1.50 (1.02–2.20)	0.039
Past/current history of stroke				
No	219/1171 (18.7)			
Yes	21/116 (18.1)	0.875		
Past/current history of coronary heart disease				
No	204/1155 (17.7)		1.00	
Yes	36/132 (27.3)	0.007	1.58 (1.02–2.43)	0.039
Past/current history of hypertension				
No	153/784 (19.5)			
Yes	86/502 (17.1)	0.284		
Past/current history of diabetes mellitus				
No	222/1200 (18.5)			
Yes	18/89 (20.2)	0.687		
Knee joint pain in the last year				
No pain	102/693 (14.7)		1.00	
Pain without medical consultation	69/330 (20.9)		1.34 (0.94–1.91)	0.108
Pain requiring medical consultation	70/284 (24.7)	0.001	1.51 (1.05–2.19)	0.028
Past/current history of cancer				
No	233/1244 (18.7)			
Yes	7/44 (15.9)	0.637		

Abbreviations: OR, odds ratio; CI, confidence interval.

^a^Due to missing values, the total for all stratified subgroups may not equal 1320.

^b^On chi-square test.

^c^After adjustment for age group, sex, and factors associated with tinnitus in univariate analyses (*P* < 0.2). The 1236 participants who provided complete responses regarding these factors were included in this multivariate-adjusted analysis.

## DISCUSSION

In the present study of community-dwelling Japanese aged 65 years or older, approximately 1 in 5 had tinnitus; however, there were no statistically significant differences according to age group or sex. Hearing difficulty, depressed feeling, prescribed medication, past/current history of coronary heart disease, and knee joint pain requiring medical consultation were associated with tinnitus. To the best of our knowledge, this is the first community-based study of the prevalence and factors associated with tinnitus in an older non-Western population.

Few community-based studies of older populations have estimated the prevalence of tinnitus. The prevalence reported by the Epidemiology of Hearing Loss Study (Beaver Dam, Wisconsin, US)^4^ was 10.1% among those in their 60s, 8.7% among those in their 70s, and 5.5% among those over 80 years. In another large community-based study in Australia (the Blue Mountain Hearing Study),^5^ tinnitus was present in 32.7% of those in their 60s, 30.5% of those in their 70s, and 25.4% of those over 80 years. The prevalence found in the present study was approximately halfway between those reported in these 2 large studies, and closer to that of a Swedish study of a random sample of residents of Gothenburg aged 20 to 80 years, in which 20.3% of those in their 60s and 21.3% of those in their 70s had tinnitus.^8^ As there is no standard definition of tinnitus, direct comparison of the prevalences reported in these studies is difficult. In the present study, we observed no statistically significant age-related increase or sex difference in tinnitus prevalence. Since there is a clear relationship between tinnitus and hearing impairment,^2^^,^^11^ we expected that the prevalence trend for tinnitus would be similar to that of hearing impairment. In other words, we thought tinnitus prevalence would increase with age and be higher in men than in women.^22^^,^^23^ However, our results were compatible with those of the 2 abovementioned large community-based studies conducted in the United States^4^ and Australia,^5^ which found no sex differences or clear age trends with respect to prevalence. This suggests that factors other than hearing impairment are involved in the prevalence of tinnitus. A possible explanation for the plateauing of tinnitus prevalence in older adults is that they gradually come to accept tinnitus as part of the aging process.^4^^,^^24^

Although there may have been unexamined confounding factors such as noise exposure and history of ear diseases, we found that hearing difficulty, depressive mood, and past/current history of coronary heart disease were associated with tinnitus. These 3 factors are potentially modifiable factors of tinnitus that have been reported as risk factors in earlier studies,^1^^,^^2^^,^^4^^,^^13^ which suggests that tinnitus may be at least partially preventable. The alleviation of hearing difficulty with hearing aids or cochlear implantation is reported to help some people with tinnitus.^2^^,^^11^ Because tinnitus is closely associated with depression,^25^^,^^26^ antidepressant drugs are often selected for tinnitus treatment.^2^

The association between prescribed medication and tinnitus is plausible because tinnitus is a commonly reported side effect of many drugs, including salicylates, nonsteroidal anti-inflammatory drugs (NSAIDs), aminoglycoside antibiotics, loop diuretics, and chemotherapy agents, among many others.^2^^,^^27^ Therefore, we hypothesized that the observed association between knee joint pain requiring medical consultation and tinnitus might be due to the use of salicylates or NSAIDs, which are often prescribed for knee joint pain. Clinically, tinnitus has been reported as a side effect of NSAIDs in patients with osteoarthritis and rheumatoid arthritis.^28^^–^^30^ However, in the present study, knee joint pain requiring medical consultation was independently associated with tinnitus in the multivariate-adjusted model including prescribed medication. Another possible explanation for this association is the existence of factors common to knee joint pain and tinnitus, such as obesity.^1^^,^^31^ Depression is also a risk factor for both knee joint pain and tinnitus^25^^,^^26^^,^^32^; however, in this study, the association between knee joint pain and tinnitus remained even after adjusting for depressive mood. Thus, at present it is not possible to draw any clear conclusions regarding the observed association between knee joint pain requiring medical consultation and tinnitus. Further epidemiological studies are needed so that more detailed information can be collected on potential risk factors such as obesity and the contents of prescribed medication.

The strengths of our study are that it is highly representative of the target population and that it presents the first evidence of community-based prevalence of tinnitus and associated factors in an older non-Western population. Despite these strengths, the present study has some limitations. First, this study used cross-sectional data, which provide no evidence of causality in the relationship between tinnitus and its associated factors. Indeed, we cannot rule out the possibility of reverse causality. Second, because tinnitus was defined based on subjects’ recall, misclassification of tinnitus might have occurred. However, as such misclassification would likely be nondifferential with respect to associated factors, the association between these factors and tinnitus would be underestimated. Regarding depressive mood, we cannot exclude the possibility of differential misclassification, ie, subjects with depressive mood may have been more likely to recall tinnitus than subjects without a depressive mood. Finally, we were unable to evaluate the dose–response relations between associated factors and tinnitus, because only a small number of participants had severe tinnitus.

In conclusion, our study of the prevalence and factors associated with tinnitus relied on data that were highly representative of our target population. The results showed that, as in older adults in Western countries, tinnitus is a common symptom in older Japanese. Hearing difficulty, depressive mood, prescribed medication, past/current history of coronary heart disease, and knee joint pain requiring medical consultation were associated with tinnitus in this population. Further longitudinal study of this topic is therefore needed.
